# Subacute Cognitive Impairment in Individuals With Mild and Moderate COVID-19: A Case Series

**DOI:** 10.3389/fneur.2021.678924

**Published:** 2021-08-04

**Authors:** Aline de Moura Brasil Matos, Flavia Esper Dahy, João Victor Luisi de Moura, Rosa Maria Nascimento Marcusso, Andre Borges Ferreira Gomes, Fernanda Martins Maia Carvalho, Gustavo Bruniera Peres Fernandes, Alvina Clara Felix, Jerusa Smid, Jose Ernesto Vidal, Norberto Anizio Ferreira Frota, Jorge Casseb, Ava Easton, Tom Solomon, Steven S. Witkin, Camila Malta Romano, Augusto César Penalva de Oliveira, NeuroCovBR Study Group

**Affiliations:** ^1^Faculdade de Medicina, Instituto de Medicina Tropical, Universidade de São Paulo, São Paulo, Brazil; ^2^Instituto de Infectologia Emilio Ribas, São Paulo, Brazil; ^3^Hospital Geral de Fortaleza, Serviço de Neurologia, Fortaleza, Brazil; ^4^Programa de Pós-Graduação em Ciências Médicas, Universidade de Fortaleza, Fortaleza, Brazil; ^5^Hospital Israelita Albert Einstein, São Paulo, Brazil; ^6^Encephalitis Society, Malton, United Kingdom; ^7^Department of Clinical Infection, Microbiology and Immunology, University of Liverpool, Liverpool, United Kingdom; ^8^National Institute for Health Research Health Protection Unit in Emerging and Zoonotic Infections, Institute of Infection, Veterinary and Ecological Sciences, University of Liverpool, Liverpool, United Kingdom; ^9^Walton Centre National Health Service Foundation Trust, Liverpool, United Kingdom; ^10^Weill Cornell Medicine, Department of Obstetrics and Gynecology, New York, NY, United States; ^11^Faculdade de Medicina, Hospital das Clinicas, Universidade de São Paulo, São Paulo, Brazil

**Keywords:** COVID-19, Encephalopathies, executive function, viral infection, cognitive impairment

## Abstract

**Background:** Previous reported neurologic sequelae associated with SARS-CoV-2 infection have mainly been confined to hospital-based patients in which viral detection was restricted to nasal/throat swabs or to IgM/IgG peripheral blood serology. Here we describe seven cases from Brazil of outpatients with previous mild or moderate COVID-19 who developed subacute cognitive disturbances.

**Methods:** From June 1 to August 15, 2020, seven individuals 18 to 60 years old, with confirmed mild/moderate COVID-19 and findings consistent with encephalopathy who were observed >7 days after respiratory symptom initiation, were screened for cognitive dysfunction. Paired sera and CSF were tested for SARS-CoV-2 (IgA, IgG ELISA, and RT-PCR). Serum and intrathecal antibody dynamics were evaluated with oligoclonal bands and IgG index. Cognitive dysfunction was assessed by the Mini-Mental State Examination (MMSE), Montreal Cognitive Assessment (MoCA), and the Clock Drawing Test (CDT).

**Results:** All but one of our patients were female, and the mean age was 42.6 years. Neurologic symptoms were first reported a median of 16 days (IQR 15–33) after initial COVID-19 symptoms. All patients had headache and altered behavior. Cognitive dysfunction was observed mainly in phonemic verbal fluency (MoCA) with a median of six words/min (IQR 5.25–10.75) and altered visuospatial construction with a median of four points (IQR 4–9) (CDT). CSF pleocytosis was not detected, and only one patient was positive for SARS-Co

**Conclusions:** A subacute cognitive syndrome suggestive of SARS-CoV-2-initiated damage to cortico-subcortical associative pathways that could not be attributed solely to inflammation and hypoxia was present in seven individuals with mild/moderate COVID-19.

## Introduction

Severe acute respiratory syndrome coronavirus-2 (SARS-CoV-2), the virus that causes coronavirus disease 2019 (COVID-19), was first reported in December 2019 in Hubei province, China (1). By May 2021, more than 160 million people were infected by this virus worldwide and over 3.4 million died in the most severe pandemic since the 1918 Spanish Flu (2). From the very beginning, non-specific neurological symptoms such as headache, confusion, and dizziness were reported during the acute phase of COVID-19 (3). Alterations in mental status and behavior were, at first, attributed to a direct infection by SARS-CoV-2 or to the prolonged use of neuromuscular blockers and/or sedative medications (4).

Researchers from the United Kingdom described the first large multicenter investigation of neurologic parameters in SARS-CoV-2 infection (4), followed by studies from France and Italy (5, 6). Altered mental status was the second most frequent manifestation noted. Most reported cases were hospital-based and had severe COVID-19, and SARS-CoV-2 detection was restricted to nasal/throat swabs or to IgM/IgG peripheral blood serology. During the first COVID-19 wave, restricted evidence was presented of neurological manifestations associated with mild or moderate SARS-CoV-2 infection in young patients (4). Viral dynamics in the central nervous system (CNS) was also poorly investigated.

To address the issue of possible neurological manifestations in mild, moderate, severe, and critical COVID-19 infections, the NeuroCovBR study group assembled a multicenter cohort of six neurology reference centers from three different Brazilian administrative regions. After the first included patients, we hypothesized that COVID-19-associated encephalopathic conditions differed from the binomial sepsis—encephalopathy. In support of this possibility, we now describe seven patients from our cohort seen on an outpatient basis who developed encephalopathy and cognitive impairment >7 days after their first mild/moderate manifestations of COVID-19. None had severe illness, were prescribed medications, or exhibited metabolic dysfunctions that could link their clinical presentation to a classic diagnosis of delirium.

## Methods

### The NeuroCovBR

A consortium of investigators from six regional SARS-CoV-2 pandemic epicenters located in the Southeast, Northeast, and Federal Districts of Brazil (NeuroCovBR) participates in this prospective cohort neurological study. Included patients must present with possible, probable, or confirmed SARS-CoV-2 meningitis, encephalitis, myelitis, CNS vasculitis, acute disseminated encephalomyelitis, Guillain–Barré syndrome, or other acute neuropathies as defined provisionally by Ellul (7). COVID-19 was defined and classified as mild, moderate, severe, or critical according to the World Health Organization (8, 9).

As a brief description of the study design, patients are referred to the study sites' neurologists by non-specialists (i.e., general practitioners, infectious diseases doctors, intensive care physicians) if any of the above syndromes is suspected as inpatient consultant or outpatient visits. First neurological symptoms must occur within 60 days of the first COVID-19 symptoms, regardless of COVID-19 severity. Demographics, clinics, laboratory, MRI, and electrophysiology tests results are collected and stored at an electronic database designed for this study at REDCap. Additional virology tests are done as described below.

This study is meant to last for 2 years, and the first enrollment occurred by June 1, 2020. It was approved by the Universidade de São Paulo ethics committee (CAAE: 31378820.1.1001.0068) and study site ethics committees. All patients provided written informed consent, and their personal information is protected according to ethical procedures.

### Cognitive Impaired Patients

As provisionally defined by Ellul (7) and adopted in our study, SARS-CoV-2 encephalitis is divided into four levels: level 1, encephalitis, level 2, possible encephalitis, level 3, encephalopathy, and level 4, possible encephalopathy. At level 4, we have patients with “suspected encephalopathy (an alteration in consciousness, cognition, personality or behavior) with no other diagnosis apparent, but does not fulfill level 3 criteria,” and at level 3 we additionally have “acute or sub-acute (<4 weeks) alteration in consciousness, cognition (including delirium or coma), personality or behavior persisting for more than 24 h” and “absence of an alternative diagnosis for symptoms” (7).

Levels 3 and 4 of SARS-CoV-2 encephalitis (or SARS-CoV-2 encephalopathy) were the main cause of patients' inclusion in the first 45 days of NeuroCovBR enrollment. By that time, we expected this clinical condition to be restricted to patients with severe COVID-19 resembling manifestations of septic encephalopathy similar to other infectious conditions. However, a considerable subset of mild and moderate COVID-19 patients presented this feature. Due to this atypia, here we describe these patients in advance.

### SARS-CoV-2 Encephalopathy Patients

We identified seven NeuroCovBR outpatients patients seen from June 1, 2020, to August 15, 2020, with previous confirmed mild or moderate COVID-19, as defined by the World Health Organization (8), who presented with SARS-CoV-2 encephalopathy at least 7 days after manifestation of COVID-19-related symptoms (8).

Exclusion criteria for this report were previous use of medications known to cause cognitive dysfunction (i.e., neuromuscular blockers, sedatives, analgesics, antipsychotics), previous neurologic or psychiatric conditions (i.e., Alzheimer's disease, Parkinson's disease, stroke, epilepsy), and acute metabolic dysfunction (i.e., acute or acute-on-chronic renal failure, altered sodium, altered potassium, hypoglycemia, hyperglycemia).

### Cognitive Screening

All subjects included were screened for cognitive dysfunction using the Mini-Mental State Examination (MMSE), Montreal Cognitive Assessment (MoCA), and/or Clock Drawing Test (CDT). During this first period of our study, we were only able to perform cognitive screening tests since neuropsychology attendance was suspended by sanitary measures. For the MMSE, the cutoffs for cognitive impairment were 20 points for illiterates, 25 points for 1–4 years of schooling, 26 points for 5–8 years schooling, and 28 points for 9 or more years of schooling, as reported previously for Brazilian subjects (10). For the MoCA test, 25 points was chosen as the cutoff for mild cognitive impairment (11). Patients with <5 schooling years were not submitted to this test due to low accuracy in detecting dementia in this population (12). For the CDT, we used the algorithm method proposed by Mendes-Santos (13), scoring drawn clocks from 1 to 10 points. As an evaluation of visuospatial function, scores lower than 9 were considered as evidence of visuospatial impairment. For each of the three tests, more important than cutoff values was the analysis of the affected cognitive domains. This may be present in cases where the MMSE analysis was normal while the MoCA and CDT results indicated impairments.

### Laboratory Procedures

Paired serum and cerebrospinal fluid (CSF) were tested for SARS-CoV-2 IgA and IgG antibodies by a commercial ELISA kit (Euroimmun, Lubeck, Germany) and for SARS-CoV-2-specific RNA by RT-PCR (RealStar RT-PCR kit, Altona Diagnostics, Hamburg, Germany), according to the manufacturers' directions. Blood was tested for cell count, electrolytes, glucose, renal and liver function, C-reactive protein, and D-dimer, by routine laboratory protocols. Routine CSF analyses included cytologic evaluation and biochemical evaluation, lactate measurement, and screening for common local infectious diseases.

Serum and intrathecal samples were analyzed for oligoclonal bands (OCBs), i.e., subfractions of IgG that are encountered in individuals with autoimmune or infectious disorders (14, 15), by isoelectric focusing (Hydragel 9 CSF isofocusing, Sebia, Paris, France). Results were classified as pattern 1—no OCB detected, pattern 2—OCB in CSF only, pattern 3—identical OCBs in CSF and serum with additional OCBs in CSF, pattern 4—identical OCBs in CSF and serum, and pattern 5—ladder OCBs. The IgG index, a measurement of IgG production in serum or CSF, was calculated as [IgG (CSF) × albumin (serum)/IgG (serum) × albumin (CSF)] × 100. Albumin and IgG levels were determined by nephelometry. OCB patterns 2 and 3 and an IgG index >0.7 are indicative of intrathecal antibody production.

### Neuroimage and EEG

All patients were submitted either to a brain MRI or to CT scan, based on analysis of the attending physician and hospital availability. The findings were evaluated by a radiologist with training in neuroradiology. EEG was performed in all patients according to international recommendations (16).

### Statistics

Continuous data are summarized as median and interquartile range (IQR). Categorical data are presented as counts and percentages. Data were analyzed using SPSS version 25.0.

## Results

**Figure 1:**
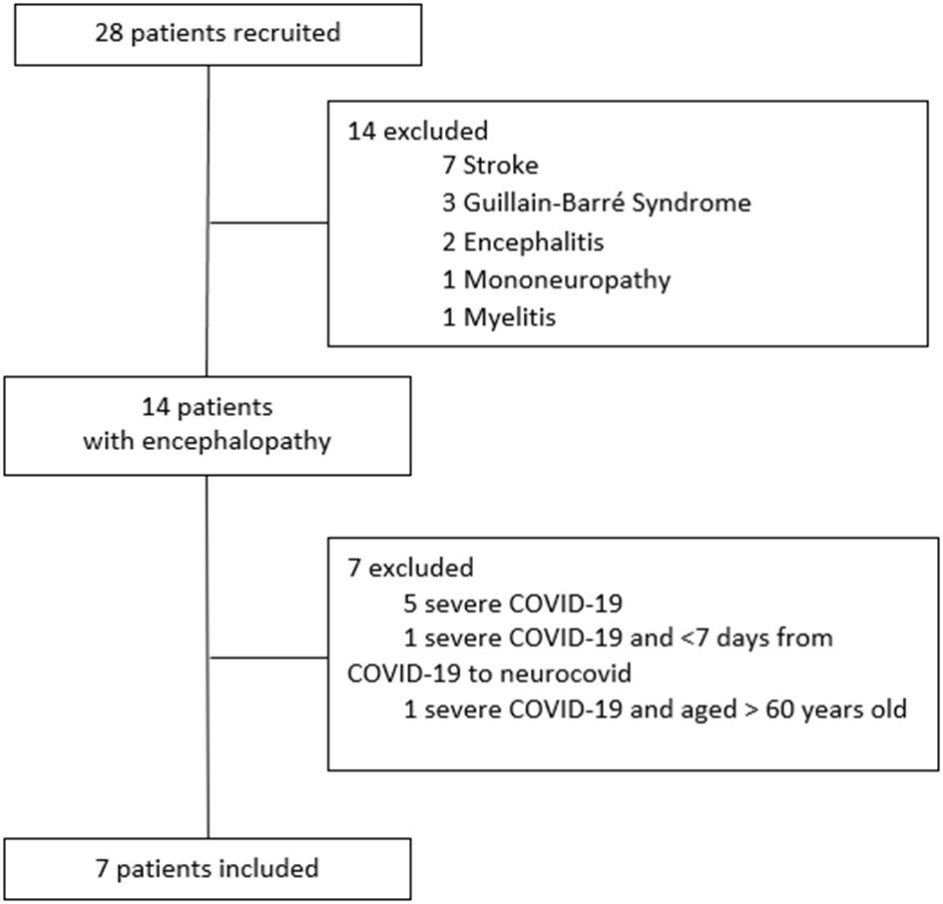
Patients flow-chart. neurocovid, neurological manifestations of COVID-19. Encephalopathy was defined according to the Ellul et al.

During the inclusion window, seven out of 28 patients recruited (25%) filled inclusion criteria (Figure 1). Characteristics of the study population are described in Table 1. Six of the seven subjects (85.7%) were female, and the median age (IQR) was 44 (39–47) years. Five (71.4%) had comorbidities, two each with hypertension or diabetes and one with asthma. Five were diagnosed with mild COVID-19, and two had a moderate disease based primarily on the presence or absence of symptoms of mild pneumonia. Neurologic symptoms developed at a median of 16 (15–33) days after initial COVID-19 manifestations. All patients had headache and altered behavior (reported as mild irritability or aggressiveness). Five also reported periods of time/space confusion lasting <24 h, and six had difficulties in going to sleep or remaining asleep.

**Table 1:** Clinical and laboratory characteristics of patients with neurological symptoms associated with COVID-19.

**Patient**	**Age**	**Sex**	**Comorbidities**	**COVID-19 severity**	**Days from COVID-19 to neurologic symptoms**	**Neurologic symptoms**	**MMSE**	**MoCA**	**CDT**	**CSF PCR/ IgA/IgG**	**Serum PCR/ IgA/IgG**	**OCB pattern**	**IgG Index**
1^*^	30	F	Asthma	mild	15	Headache, altered behavior, sleep disturbance, T/S confusion, seizure	23/30	–	4/10	/ – /– / – /	/ – /– / + /	1	0.43
2^*^	47	F	HT	mild	16	Headache, altered behavior, sleep disturbance, T/S confusion	30/30	24/30	9/10	/ + /– / – /	/ – /– / + /	NA ^‡^	0.44
3^*^	44	F	–	mild	33	Headache, altered behavior, sleep disturbance, T/S confusion	–	–	4/10	/ – /– / – /	/ – /+ / + /	NA ^‡^	NA^‡^
4^*^	39	F	–	mild	52	Headache, altered behavior, sleep disturbance	–	28/30	9/10	/ – /– / – /	/ – /– / + /	1	0.5
5^*^	45	F	DM	mild	9	Headache, altered behavior, sleep disturbance, T/S confusion, altered level of conscience	29/30	–	4/10	/ – /– / – /	/ – /+ / + /	1	0.46
6^*^	53	F	HAS	moderate	15	Headache, altered behavior, sleep disturbance	23/30	–	4/10	/ – /– / – /	/ – /+ / + /	1	0.52
7^†^	40	M	DM	moderate	26	Headache, altered behavior, T/S confusion, altered level of conscience	17/30	9/30	1/10	/ – /– / – /	/ – /– / + /	1	0.44

*MMSE, mini-mental state examination; MoCA, montreal cognitive assessment; CDT, clock drawing test; OCB, oligoclonal bands; HT, hypertension; DM, diabetes mellitus; T/S, time and space; NA, not applied*.

* *>8 scholar years;*

† *<4 scholar years;*

‡ *not enough sample to proper perform the test; OCB patterns: 1 no OCBs seen, 2 OCBs in CSF only, 3 identical OCBs in CSF and serum with extra in CSF, 4 identical OCBs in both, 5 ladder OCBs*.

*IgG index cut off < 0.7*.

*CDT points range from 1 to 10*.

*COVID-19 severity is defined according to WHO*.

Table 1 lists the laboratory test results. Cognitive dysfunction was observed in all patients, mainly in phonemic verbal fluency (MoCA) with a median of six words/min (IQR 5.25–10.75) and visuospatial construction with a median of four points (IQR 4–9) in the CDT. Patients 1 to 5 had normal brain MRI scans, and patients 6 and 7 had normal brain CT scans. In addition, routine blood and CSF analyses were all within normal limits.

**Figure 2:**
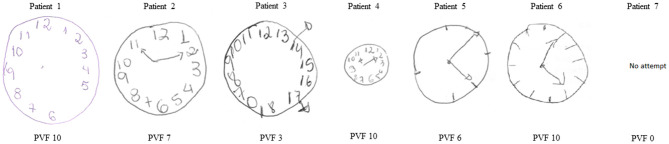
Clock drowing test and phnemic verval fluency from patients with neurological symptoms associated with COVID-19. PVF, phonemic verbal fluney. PVF cut off ≥11 words in a minute.

Only one patient (number 2) was positive for SARS-CoV-2 by PCR, and all seven were negative for IgG and IgA anti-SARS antibodies. In the serum, all were PCR-negative, including the patient who was positive for SARS-CoV-2 in her CSF. Three patients were positive for IgA antibodies, and all seven had IgG anti-SARS-antibodies. The one patient positive for SARS by PCR in her CSF had a score of 30/30 on her MMSE and 9/10 on her CDT test. The results of the CDT test on all patients are shown in Figure 2. Of the four patients who had a score of 4/10 on their CDT test, three were IgA anti-SARS antibody-positive in their serum. There were no apparent associations between the PCR and antibody findings, the three neurology test results, and the occurrence of specific symptoms. None of the patients had an OCB pattern indicative of autoantibodies, and all had an IgG index in the normal range.

## Discussion

We describe seven patients with subacute SARS-CoV-2 encephalopathy and cognitive impairment associated with mild to moderate COVID-19. Their cognitive dysfunction was predominantly a dysregulation of executive activities that are associated with frontal lobe damage. Dysexecutive syndrome typically encompasses emotional, motivational, and behavioral symptoms, as well as cognitive deficits (17–19). There were no signs of intrathecal antibody production or blood–brain-barrier disruption, and MRI, EEG, and CSF findings were unremarkable. These observations are consistent with the existence of a syndrome related to SARS-CoV-2-induced damage to cortico-subcortical associative pathways.

SARS-CoV-2 is an RNA virus, and as a general rule RNA viruses remain in the circulation for only a limited time period. By the time we designed NeuroCovBR, we employed techniques for direct and indirect viral screening to maximize our chances to identify evidence of virus within the CNS. However, SARS-CoV-2 RNA was detected in only one of our patients, and no atypical OCB patterns or an abnormal IgG index was seen. Nevertheless, cognitive dysfunction in each of the seven cases was evident.

Other examples of virus-induced neurological impairment, such as HIV-Associated Neurocognitive Disorder (HAND) and Hepatitis C neurocognitive impairment (20, 21), exhibit similarities with SARS-CoV-2 encephalopathy. In those other infections, cognitive disturbances were not attributable only to direct virus-induced neurological damage. Instead, they were a consequence of a persistent subclinical inflammatory state resulting from the host's attempts at viral clearance as well as to viral-induced dysfunctions in neurotransmitter receptors that were detectable by SPECT but untraceable in routine MRI, CSF, or EEG (20, 22). HIV, for example, is not neurotropic but resides within lymphocytes that, acting as Trojan horses, can cross the blood–brain barrier and induce pro-inflammatory cytokines in the brain parenchyma causing secondary and progressive damage. The infected lymphocyte Trojan horse mechanism can also disrupt neuronal communication by inducing an environment rich in reactive oxygen species that results in both neuronal dysfunction and cell death (6). This mechanism is also feasible for SARS-CoV-2 infection, since the virus successfully infects lymphocytes (23). Another possibility for invasion is by infection of epithelial cells that express the angiotensin-converting enzyme 2 (ACE 2) receptor. SARS-CoV-2 binding to the ACE2 receptor is the major mechanism for viral entry into cells (7).

Hypoxia and pro-inflammatory cytokines are possible contributory mechanisms in SARS-CoV-2 encephalopathy (24). The analysis of pro-inflammatory cytokines along with neuronal biomarkers in patients with severe COVID-19 resembles alterations of the immune effector cell-associated neurotoxicity syndrome (ICANS), a neuropsychiatry syndrome related to chimeric antigen receptor (CAR) T cell therapy (25, 26). ICANS is associated with a cytokine release syndrome (CRS) (27) secondary to CAR-T cell therapy, and a plausible SARS-CoV-2 encephalopathy mechanism could be a COVID-19-induced CRS (28, 29). However, considering that our patients had mild/moderate COVID-19, the hypothesis of an immune-mediated neurotoxicity ICANS-like is not as strong as in patients with severe COVID-19; nevertheless, a mild persistent immune-mediated inflammatory state (considering that neurological manifestations occurred in median 16 days) might lead to SARS-CoV-2 delayed cognitive disturbances as in HAND or Hepatitis C neurocognitive impairment. This time lapse between the first COVID-19 symptoms and the first neurological symptoms also resembles the pathophysiological mechanisms of autoimmune induction present for instance in viral post-infectious polyradiculoneuritis (7).

It can be argued that neuropathology studies were not able to identify modifications caused by SARS-CoV-2 invasion beyond nonspecific signs of inflammation and hypoxia (24, 30). However, a prior MRI-based study observed microstructural damage in the cerebral cortex with possible neurogenesis in frontal-subcortical pathways independent of COVID-19 severity (31). Thus, our findings most likely were not solely due to inflammation or hypoxia, since those manifestations were not major attributes in our patients. We must also consider the possibility of a specific SARS-CoV-2-induced mechanism resulting in CNS damage not identified by gross pathological analysis and that can be independent of COVID-19 severity.

Dysexecutive function is characteristic of disruptions in frontal-subcortical circuits, large neuronal circuits that originate in the frontal cortex but spread to many different cerebral areas such as the striatum and thalami (32). Considering our patients' impairments in verbal fluency, poor constructional strategies, and behavior alterations, the dorsolateral prefrontal circuit, the anterior cingulate cortex, and the lateral orbitofrontal circuit are likely the most affected regions in SARS-CoV-2 encephalopathy. These circuits have been associated with different types of subcortical infectious dementia and hypothesized as affected in COVID-19 (32, 33).

Our study has limitations. We lack data on the time course of patients' recovery from COVID-19 and on long-term follow-up of cognitive alterations. Although none of our patients were oxygen-dependent and D-dimer was unremarkable as CSF lactate, we cannot definitively rule out an underlying mechanism of brain microcapillary dysfunction associated with brain tissue hypoxia and neuroglycopenia in sepsis. The observation that six of our seven subjects were female suggests a possible gender bias in SARS-CoV-2-related adverse neurological sequelae. However, this finding may be due solely to our small sample size and additional studies are needed to evaluate this further.

In conclusion, our results are compatible with subacute cognitive dysfunctions associated with mild/moderate COVID-19 that develops in patients independent of identifiable comorbidities. The dysfunctions cannot be explained solely by inflammation or hypoxia, although these effects might contribute to the observed alterations. Our findings point to the existence of a SARS-CoV-2-induced damage of cortico-subcortical associative pathways whose natural history remains unknown.

## Data Availability Statement

The original contributions presented in the study are included in the article/Supplementary Material, further inquiries can be directed to the corresponding author/s.

## Ethics Statement

The studies involving human participants were reviewed and approved by Universidade de São Paulo ethics committee (CAAE: 31378820.1.1001.0068). The patients/participants provided their written informed consent to participate in this study. Written informed consent was obtained from the individuals for the publication of any potentially identifiable images or data included in this article.

## Author Contributions

AM, CM, and AO were responsible for the study concept and design, data acquisition, analysis and interpretation of data, and critical revision of the manuscript. FD, JM, RM, AG, FC, GF, AF, JS, and JV were responsible for data acquisition, analysis and interpretation of data, and critical revision of the manuscript. JC, AE, TS, and SW were responsible for the critical revision of the manuscript. All authors contributed to the article and approved the submitted version.

## Conflict of Interest

The authors declare that the research was conducted in the absence of any commercial or financial relationships that could be construed as a potential conflict of interest.

## Publisher's Note

All claims expressed in this article are solely those of the authors and do not necessarily represent those of their affiliated organizations, or those of the publisher, the editors and the reviewers. Any product that may be evaluated in this article, or claim that may be made by its manufacturer, is not guaranteed or endorsed by the publisher.

## NeuroCovBR Study Group

Mariana Saconato, PhD, Instituto de Infectologia Emilio Ribas, São Paulo, SP, Brazil, Investigador; Jose Angelo L. Lindoso, MD, PhD, Instituto de Infectologia Emilio Ribas, São Paulo, SP, Brazil, Investigador; Rosa PSF Ferrarese, MD, Instituto de Infectologia Emilio Ribas, São Paulo, SP, Brazil, Investigador; Graziela UL Domingues, MSc, Instituto de Infectologia Emilio Ribas, São Paulo, SP, Brazil, Investigador; Jaques Sztanjbok, MD, Instituto de Infectologia Emilio Ribas, São Paulo, SP, Brazil, Investigador; Michel EJ Haziot, MD, Instituto de Infectologia Emilio Ribas, São Paulo, SP, Brazil, Investigador; Rene LM Rivero, MD, PhD, Instituto de Infectologia Emilio Ribas, São Paulo, SP, Brazil, Investigador; Lucio NA Batista, Instituto de Infectologia Emilio Ribas, São Paulo, SP, Brazil, Investigador; Cleonisio L, Rodrigues, MD, PhD, Hospital Geral de Fortaleza, Fortaleza, CE, Brazil, Principal Investigator; Isaac HM, Maia, MD, Hospital Geral de Fortaleza, Fortaleza, CE, Brazil, Investigador; Daniele M. Lima, PhD, Hospital Geral de Fortaleza, Fortaleza, CE, Brazil, Investigador; Fabricio O. Lima, MD, PhD, Hospital Geral de Fortaleza, Fortaleza, CE, Brazil, Investigador; Felipe A. Rocha, MD, Hospital Geral de Fortaleza, Fortaleza, CE, Brazil, Investigador; Tiago P. Feijo, MD, Hospital Geral de Fortaleza, Fortaleza, CE, Brazil, Investigador; Daniel G. F. Tavora, MD, Hospital Geral de Fortaleza, Fortaleza, CE, Brazil, Investigador; Karoline F. Mororo, MD, Hospital Geral de Fortaleza, Fortaleza, CE, Brazil, Investigador; Francisco Silvanei S. Gonçalves, Hospital Geral de Fortaleza, Fortaleza, CE, Brazil, Investigador; Anderson V. Paula, Universidade de São Paulo, São Paulo, SP, Brazil, Investigador; Francisco T. M. Oliveira, MD, MSc, Santa Casa de Misericórdia de São Paulo, São Paulo, SP, Brazil, Principal Investigator; Lohana A. S. Silva, MD, Santa Casa de Misericórdia de São Paulo, São Paulo, SP, Brazil, Investigador; Rodrigo M. Massaud, MD, Hospital Israelita Albert Einstein, São Paulo, SP, Brazil, Principal Investigator; Lorena S. Viana, MD, Hospital Israelita Albert Einstein, São Paulo, SP, Brazil, Principal Investigator; Marcel K. Uehara, MD, Hospital Israelita Albert Einstein, São Paulo, SP, Brazil, Investigador; Marcos V. T. Fujino, MD, Hospital Israelita Albert Einstein, São Paulo, SP, Brazil, Investigador; Thiago D. Correa, MD, PhD, Hospital Israelita Albert Einstein, São Paulo, SP, Brazil, Investigador; Alcino A. Barbosa Jr, MD, Hospital Israelita Albert Einstein, São Paulo, SP, Brazil, Investigador; Fabiana, Hirata, MD, PhD, Hospital Israelita Albert Einstein, São Paulo, SP, Brazil, Investigador; Iron, Dangoni Filho, MD, Hospital Israelita Albert Einstein, São Paulo, SP, Brazil, Investigador; Victor R. Procaci, MD, Hospital Israelita Albert Einstein, São Paulo, SP, Brazil, Investigador; Natalia M, Athayde, MD, Hospital Israelita Albert Einstein, São Paulo, SP, Brazil, Investigador; Felipe, Von Glehn, MD, PhD, Hospital Universitário de Brasília, Brasília, DF, Brazil, Principal Investigator; Raimundo N. D. Rodrigues, MD, PhD, Hospital Universitário de Brasília, Brasília, DF, Brazil, Investigador; Pedro A. L, Oliveira, MD, MSc, Hospital Universitário de Brasília, Brasília, DF, Brazil, Investigador; Marcia S. S. Neiva, MD, Hospital Universitário de Brasília, Brasília, DF, Brazil, Investigador; Luciano T. Ferreira, MD, Hospital Universitário de Brasília, Brasília, DF, Brazil, Investigador; Keila RFG. Gal, MD, Hospital Universitário de Brasília, Brasília, DF, Brazil, Investigador; Priscilla M. Proveti, MD, MSc, Hospital Universitário de Brasília, Brasília, DF, Brazil, Investigador; Leticia C. Rebello, MD, Hospital Sirio Libanes Brasília, Brasília, DF, Brazil, Principal Investigator; Pedro RP, Brandão, MD, Hospital Sirio Libanes Brasília, Brasília, DF, Brazil, Investigador; Ingrid F. Vasconcellos, MD, PhD, Hospital Sirio Libanes Brasília, Brasília, DF, Brazil, Investigador; Victor M. Caldas, MD, Hospital Sirio Libanes Brasília, Brasília, DF, Brazil, Investigador; Luciana M. Barbosa, MD, Hospital Sirio Libanes Brasília, Brasília, DF, Brazil, Investigador;
